# A practical nomogram for preoperatively predicting lateral cervical lymph node metastasis in medullary thyroid carcinoma: a dual-center retrospective study

**DOI:** 10.3389/fendo.2024.1349853

**Published:** 2024-07-26

**Authors:** Jialin Zhu, Tiantian Guo, Shuyue Guo, Luchen Chang, Jing Zhao, Xiaoqing Wang, Xi Wei

**Affiliations:** Department of Diagnostic and Therapeutic Ultrasonography, Key Laboratory of Cancer Prevention and Therapy, Tianjin Medical University Cancer Institute and Hospital, National Clinical Research Center for Cancer, Tianjin, China

**Keywords:** nomogram, medullary thyroid carcinoma, metastasis, ultrasonography, prediction

## Abstract

**Purpose:**

Lateral lymph node metastasis (LLNM) is very common in medullary thyroid carcinoma (MTC), but there is still controversy about how to manage cervical lateral lymph nodes, especially for clinically negative MTC. The aim of this study is to develop and validate a nomogram for predicting LLNM risk in MTC.

**Materials and methods:**

A total of 234 patients from two hospitals were retrospectively enrolled in this study and divided into LLNM positive group and LLNM negative group based on the pathology. The correlation between LLNM and preoperative clinical and ultrasound variables were evaluated by univariable and multivariable logistic regression analysis. A nomogram was generated to predict the risk of the LLNM of MTC patients, validated by external dataset, and evaluated in terms of discrimination, calibration, and clinical usefulness.

**Results:**

The training, internal, and external validation datasets included 152, 51, and 31 MTC patients, respectively. According to the multivariable logistic regression analysis, gender (male), relationship to thyroid capsule and serum calcitonin were independently associated with LLNM in the training dataset. The predictive nomogram model developed with the aforementioned variables showed favorable performance in estimating risk of LLNM, with the area under the ROC curve (AUC) of 0.826 in the training dataset, 0.816 in the internal validation dataset, and 0.846 in the external validation dataset.

**Conclusion:**

We developed and validated a model named MTC nomogram, utilizing available preoperative variables to predict the probability of LLNM in patients with MTC. This nomogram will be of great value for guiding the clinical diagnosis and treatment process of MTC patients.

## 1 Introduction

Medullary thyroid carcinoma (MTC) is a life-threatening disease that accounts for approximately 5%–10% of all thyroid malignancies worldwide and 13.4% of deaths from thyroid cancer are attributed to MTC (1, 2). The prominent feature of MTC is relatively slow tumor growth, but early cervical lymph node metastasis (LNM) occurs, with LLNM occurring in approximately 40.0%-66.7% of patients diagnosed with MTC for the first time (3, 4). In particular, lateral neck lymph node metastasis (LLNM) was very common in MTC. The American Thyroid Association (ATA) recommended that the curative therapy for MTC was primary tumor resection and cervical lymph node dissection (5). However, the extent of initial surgery for cervical lymph node dissection is still controversial (6), especially whether lateral lymph node dissection (LND) is necessary in MTC (7). Some surgeons prefer more proactive management, such as performing lateral LND or prophylactic dissection, to improve postoperative biochemical recovery rates. However, more aggressive prophylactic LND may increase the risk of severe neurological damage and hypoparathyroidism without significant survival benefits (8).

Preoperative imaging plays an important guiding role in the diagnosis and staging of MTC. Ultrasound (US) is the main method for evaluating lymphatic status of MTC before surgery. However, the diagnostic sensitivity for cervical lymph node metastasis (LNM) of US is only about 20%-40% (9). Many MTC patients experienced occult LLNM, which were with clinically negative (cN0) lateral cervical lymph nodes. Considering the existence of occult LLNM that is difficult to detect preoperatively, some patients undergoing thyroidectomy may already have some metastatic lymph nodes in the lateral compartment of the neck, which leading poor prognosis and high mortality. Therefore, accurately assessing the status of lateral cervical lymph node in MTC patients is all-important for choosing appropriate surgical strategy and making clinical decisions.

Serum calcitonin (Ctn) was reported to be a predictive factor for the need of prophylactic lateral lymph node dissection in MTC patients. A high preoperative Ctn level was related to the extent of LNM and poor prognosis in MTC (10). However, serum Ctn has a high false-positive rate, which had a non-specific increase in various physiological and pathological conditions (11). The 2023 European Thyroid Association (ETA) guidelines for thyroid nodule management recommends measuring Ctn in specific scenarios, such as in patients scheduled for surgery or thermal-ablation, or with nodules with indeterminate cytology or suspicious sonographic pattern, or with family history of MTC or multiple endocrine neoplasia (MEN) (12). The 2015 ATA guidelines confirmed the role of preoperative serum Ctn in determining whether lateral neck dissection should be performed in patients with cN0 and cN1a (5), but did not provide a clear threshold.

In this study, we aimed to establish a multivariate logistic regression model based on neck ultrasound examination, serum Ctn levels and other prognostic factors to predict the risk of LLNM. This model was further presented as a nomogram and provided individual recommendations for whether lateral neck lymph node dissection should be performed in MTC patients.

## 2 Materials and methods

### 2.1 Patients and datasets

We retrospectively evaluated the preoperative clinical and US features for predicting LLNM in patients with pathologically confirmed MTC surgery in Tianjin Medical University Cancer Institute and Hospital (Hospital 1) between January 2012 and March 2022 and in Binzhou Medical University Hospital (Hospital 2) between January 2017 and December 2019. Patients in Hospital 1 were randomly divided into training and internal validation datasets (3:1), while patients in Hospital 2 were defined as external testing dataset.

This retrospective study was approved by the Ethics Committee of the Tianjin Medical University Cancer Institute and Hospital, and the requirement for informed consent was waived.

### 2.2 Inclusion and exclusion criteria

**Figure 1:**
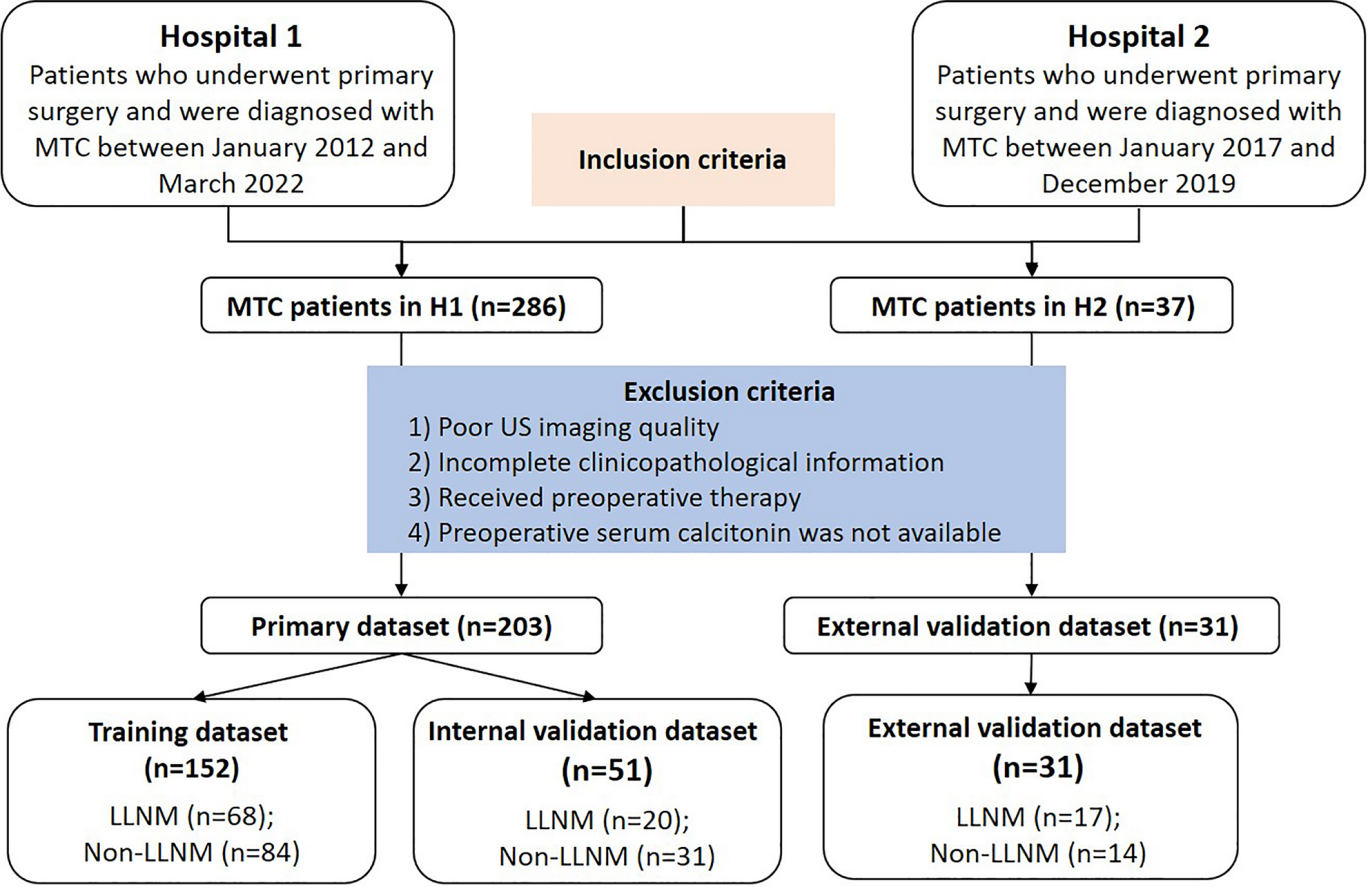
Flow chart of the patients enrolled in our study. Finally, 234 MTC patients from two centers were enrolled in this study. MTC, medullary thyroid carcinoma; LLNM, lateral lymph node metastasis; H1, hospital 1; H2, hospital 2.

Patients were enrolled based on the following criteria: 1) Pathological examinations were performed to confirm MTC with/without LLNM by surgery and fine needle aspiration cytology (FNAC). 2) Patients with clear US imaging of the thyroid. 3) Patients with complete clinical information such as clinical and US features. 4) preoperative serum Ctn measurements were available for analysis. Exclusion criteria were as follows: 1) Incomplete or unqualified ultrasound images. 2) Cases with incomplete clinicopathological information. 3) Patients who had received preoperative therapy before image acquisition. 4) Preoperative serum calcitonin was not available for analysis. All enrolled patients were followed up for at least 12 months. No suspicious lateral lymph node was seen on ultrasound and serum Ctn was not increased for patients in non-LLNM group during the follow-up period. During this study, two hospitals used the same method to detect serum Ctn. **Figure 1** illustrates the flow chart of recruitment process of the final study patients.

FNAC and surgical strategy was shown in **Supplementary Material 1**.

### 2.3 Clinical data and ultrasound images

Clinical information, results of preoperative serum Ctn testing, ultrasonic measurements and features were collected for data analysis. US characters of MTC were analyzed by experienced radiologists with more than 10 years of experience in thyroid diagnosis. The US machine included Aplio 400, 500, 800 (Toshiba Medical Systems, Tokyo, Japan) and Phillips EPIQ 5, IU 22, HD11 (Philips Healthcare, Eindhoven, The Netherlands) devices equipped with a 4.8–11 MHz, 5-12 MHz or 5–18 MHz linear array probe.

Ultrasound characteristics evaluating was shown in **Supplementary Material 2**.

### 2.4 Feature selection

We randomly allocated patients to a training dataset of 75% patients and an internal validation dataset of 25% patients in Hospital 1. Student’s independent t-test and χ2 test were applied to select the risk parameters associated with LLNM significantly. Multivariate regression analysis conducted by combining significant clinical and US variables was performed to decide the final indicators for predicting LLNM. Statistical significance was determined by a two- tailed P<0.05.

### 2.5 Construction and validation of the nomograms

Based on the multivariate analysis in the training dataset, the MTC nomogram was developed. Then, the MTC nomogram was tested by validation datasets. Calibration plots were used to validate the nomograms’ calibration. In addition, the area under the curves (AUC) of the receiver operating characteristic (ROC) curve analysis was utilized to evaluate the discriminability of the nomograms. A decision curve analysis (DCA) was conducted to evaluate the clinical net benefit of the nomogram. According to the nomogram algorithm, the predicted probability of each nodule was calculated and defined as Nomoscore. Then, the best cutoff value was determined by maximizing the Youden index. The predictive performance of the optimal cutoff value of the Nomoscore was evaluated by the AUC, sensitivity, specificity, positive predictive value (PPV), and negative predictive value (NPV). In comparison, the predictive performance of the top three important indicators was evaluated separately in the same way as the nomogram.

### 2.6 Statistical analysis

All analyses were performed using R statistical software (version 3.3.3; www.R-project.org). Two-sided p-value < 0.05 indicated statistical significance. The Mann-Whitney U test and chi-square test were separately used to compare the differences in continuous variables and categorical variables. The model predictions were assessed by sensitivity, specificity, PPV, NPV, AUC and 95% confidence interval (CI) as well as calibration curves in both the training and validation dataset. Delong test was used to compare different AUC. Calibration plot analysis was performed by bootstrapping with 1,000 replications.

## 3 Results

### 3.1 Patient characteristics

A total of 234 patients with MTC were extracted for analysis, including 152 patients (F/M = 76/76) in the training dataset, 51(F/M =35/16) in the internal validation dataset, and 31(F/M = 15/16) in the external testing dataset. As shown in **Table 1**, The mean age of patients was 48.16 ± 13.28 years for the training dataset, 49.81 ± 13.31 years for the internal testing dataset and 53.77 ± 17.09 years for the external testing dataset. The rate of LLNM in three datasets was 44.74% (68/152), 39.22% (20/51) and 54.84% (17/31), respectively.

**Table 1:** Patient characteristics of the training and testing datasets.

Characteristic	Training Dataset (%)	Internal Testing Dataset (%)	External Testing Dataset (%)
*n*	152	51	31
**Age,** mean ± SD, years	48.16 ± 13.28	49.81 ± 13.31	53.77 ± 17.09
Gender, n			
Female	76(50.00)	35(68.63)	15(48.39)
Male	76(50.00)	16(31.37)	16(51.61)
**Tumor size (cm)**	1.45 ± 0.85	1.42 ± 0.75	1.08 ± 0.78
Tumor position			
Upper pole	67(44.08)	25(49.02)	20(64.51)
Middle pole	82(53.95)	26(50.98)	11(35.48)
Lower pole	3(1.97)	0(0.00)	0(0.00)
Composition			
Mixed cystic and solid	8(5.26)	3(5.88)	2(6.45)
Solid	144(94.74)	48(94.12)	29(93.55)
Internal echo			
Hypoechoic	130(85.53)	44(86.27)	17(54.84)
Markedly hypoechoic	22(14.47)	7(13.73)	14(45.16)
Margins			
Well‐defined	87(57.24)	26(50.98)	10(32.26)
Ill‐defined	65(42.76)	25(49.02)	21(67.74)
Shape			
A/T<1	92(60.53)	29(56.86)	20(64.52)
A/T≥ 1	60(39.47)	22(43.14)	11(35.48)
Microcalcification			
No	40(26.32)	16(31.37)	10(32.26)
Yes	112(73.68)	35(68.63)	21(67.74)
Vascularization (blood flow)			
0	28(18.42)	14(27.45)	6(19.35)
1	46(30.26)	12(23.53)	2(6.45)
2	19(12.50)	9(17.65)	8(25.81)
3	59(38.82)	16(31.37)	15(48.39)
Capsule			
Not close to	72(47.37)	28(54.90)	14(45.16)
Close to	47(30.92)	13(25.49)	9(29.03)
Extrathyroidal extension	33(21.71)	10(19.61)	8(25.81)
Mulifocality			
No	110(72.37)	34(66.67)	22(70.97)
Yes	42(27.63)	17(33.33)	9(29.03)
Hashimoto thyroiditis			
Negative	114(75.00)	36(70.59)	23(74.19)
Positive	38(25.00)	15(29.41)	8(25.81)
**Serum Ctn (pg/mL, median, IQR)**	769(100-1348)	612(51-946)	1008(157-2000)
LLNM status in pathology			
Non-LLNM	84(55.26)	31(60.78)	14(45.16)
LLNM	68(44.74)	20(39.22)	17(54.84)

A/T<1, wider-than-tall; A/T≥ 1, taller than wide; Ctn, calcitonin; LLNM, lateral lymph node metastases.

### 3.2 Univariate and multivariate analysis of LLNM variables

**Figure 2:**
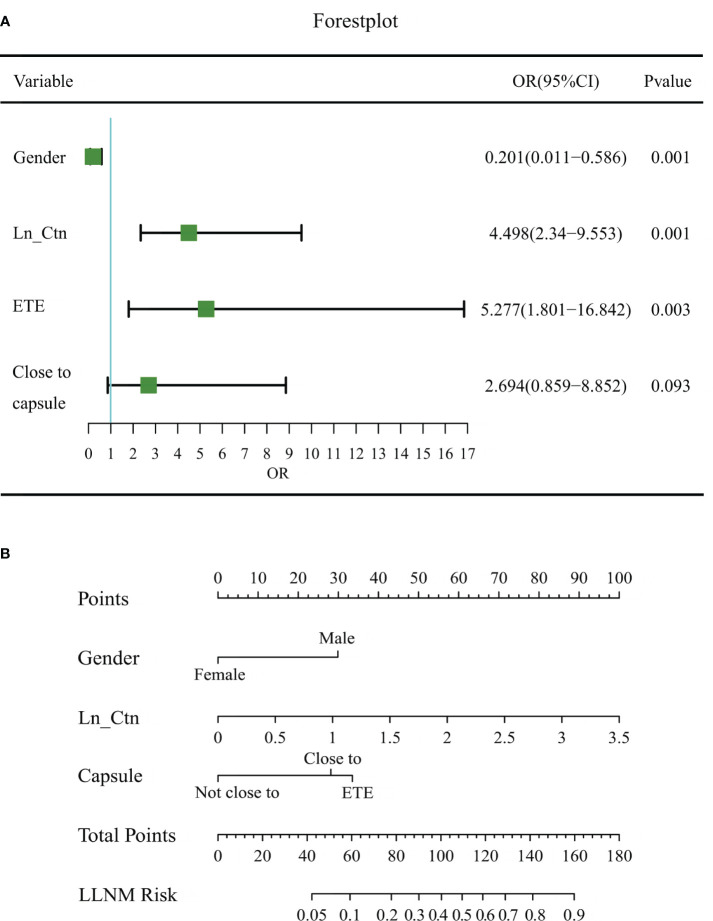
Forest plot of risk factors and the MTC nomogram for estimating the risk of LLNM in MTC. **(A)** Forest plot of risk factors in multivariable logistic regression analysis for LLNM. **(B)** The proposed nomogram based on preoperative data for assessing the risk of LLNM in MTC patients. OR, odds ratio; LN_Ctn, natural logarithm of serum calcitonin value; ETE, extrathyroidal extension; LLNM, lateral lymph node metastasis.

In the training dataset, six predictors were significantly different in the LLNM and non-LLNM groups by univariate analysis, which were gender (male), composition, microcalcification, vascularization, the relationship with the thyroid capsule and serum Ctn (**Table 2**). Multivariate logistics regression analysis identified gender (male), the position in relation to the thyroid capsule and serum Ctn as independent predictive risk factors for LLNM. Then, multivariate regression analysis was applied to construct a MTC nomogram for predicting LLNM based on these risk predictors (**Table 3**, **Figure 2A**).

**Table 2:** Patient characteristics of the MTC with LLNM and MTC without LLNM groups in the training dataset.

Characteristic	Non-LLNM group (%)	LLNM group (%)	*p*
*n*	84	68	
**Age** (years)			0.6494
≤55	54(64.28)	47(69.12)	
>55	30(35.71)	21(30.88)	
**Gender, n**			**0.001939**
Female	52(61.90)	24(35.29)	
Male	32(38.10)	44(64.71)	
**Tumor size (cm)**			0.07179
≤1	22(26.19)	14(20.59)	
>1 and ≤2	30(35.71)	16(23.53)	
>2 and ≤3	28(33.33)	28(41.18)	
>3	4(4.76)	10(14.70)	
**Tumor position**			0.0991
Upper pole	35(41.67)	32(47.06)	
Middle pole	49(58.33)	33(48.53)	
Lower pole	0 (0.00)	3(4.41)	
**Composition**			**0.02449**
Mixed cystic and solid	8(9.52)	0(0.00)	
Solid	76(90.48)	68(100.00)	
**Internal echo**			0.1212
Hypoechoic	68(80.95)	62(91.18)	
Markedly hypoechoic	16(19.05)	6(8.82)	
**Margins**			0.2593
Well‐defined	52(61.90)	35(51.47)	
Ill‐defined	32(38.10)	33(48.53)	
**Shape**			0.2222
A/T<1	55(65.48)	37(54.41)	
A/T≥ 1	29(34.52)	31(45.59)	
**Microcalcification**			**0.006155**
No	30(35.71)	10(14.71)	
Yes	54(64.29)	58(85.29)	
**Vascularization (blood flow)**			**0.0374**
0	19(22.62)	9(13.24)	
1	22(26.19)	24(35.29)	
2	6(7.14)	13(19.12)	
3	37(44.05)	22(32.35)	
**Capsule**			**<0.0001**
Not close to	54(64.29)	18(26.47)	
Close to	17(20.24)	30(44.12)	
Extrathyroidal extension	13(15.48)	20(29.41)	
**Mulifocality**			0.1759
No	65(77.38)	45(66.18)	
Yes	19(22.62)	23(33.82)	
**Hashimoto thyroiditis**			0.572
Negative	61(72.62)	53(77.94)	
Positive	23(27.38)	15(22.06)	
**Serum Ctn (pg/mL, median, IQR)**	521(30-992)	1076(274-2000)	**<0.0001**

LLNM, lateral lymph node metastases; A/T<1, wider-than-tall; A/T≥ 1, taller than wide; Ctn, calcitonin. Bold values highlight P values are less than 0.05.

**Table 3:** Risk factors for LLNM in the training dataset.

Clinical variable	Estimate	Std. Error	z value	p value
**Gender**	-1.60221	0.46602	-3.438	**0.000586**
**Ln_Ctn**	1.50361	0.35582	4.226	**<0.0001**
**Composition**	17.77591	1276.3692	0.014	0.988888
**Mircocalcification**	0.88073	0.56364	1.563	0.118149
**Extrathyroidal extension**	1.66327	0.56562	2.941	**0.003276**
**Close to Capsule**	0.99116	0.59036	1.679	0.093169
**Vascularization 1**	0.03818	0.66445	0.057	0.95418
**Vascularization 2**	1.30175	0.89358	1.457	0.145178
**Vascularization 3**	-0.97984	0.69625	-1.407	0.159339

LLNM, lateral lymph node metastases; LN_Ctn, natural logarithm of serum calcitonin value; Vascularization 1, Adler grade of blood flow was 1; Vascularization 2, Adler grade of blood flow was 2; Vascularization 3, Adler grade of blood flow was 3. Bold values highlight P values are less than 0.05.

### 3.3 Construction and validation of the nomogram

The MTC nomogram was established based on these three indicators (**Figure 2B**). The nomogram scored 30 for male, 28 for close to capsule, and 33 for break through the thyroid capsule. Because the logistic model requires a linear relationship between the continuous independent variable and the logit conversion value of the dependent variable, the value of serum calcitonin has undergone a natural logarithmic conversion, expressed as LN_Ctn. The final nomoscore cutoff for positivity is 110 with the corresponding probability of LLNM is 0.50.

**Figure 3:**
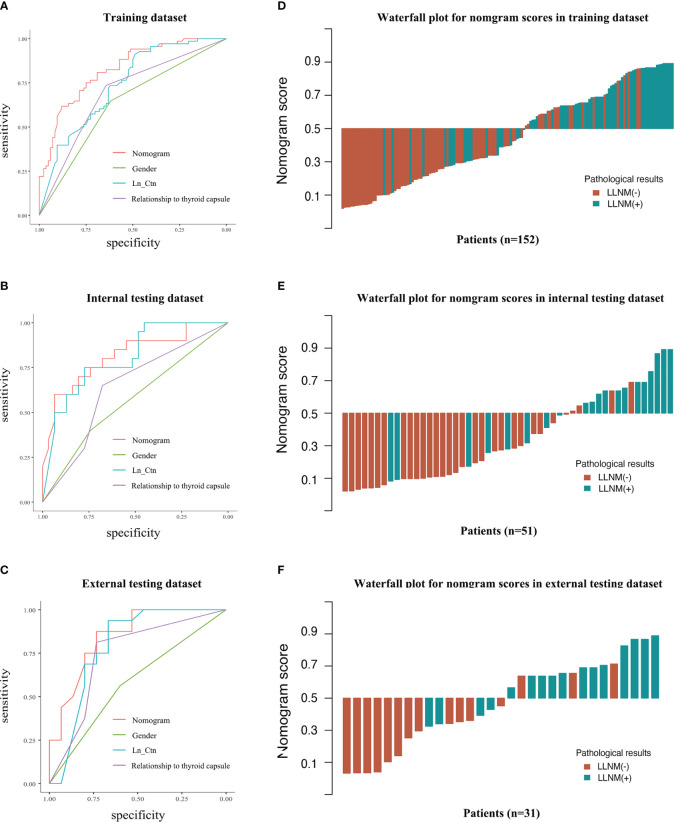
Predictive performance of the MTC nomogram and other features in discrimination of LLNM and non-LLNM in three datasets. ROC curves of MTC nomogram compared to other three features in the training dataset **(A)**, internal testing dataset **(B)** and external testing dataset **(C)**. The waterfall figures show the data distribution **(D–F)**. LN_Ctn, natural logarithm of serum calcitonin value; LLNM, lateral lymph node metastasis.

The performance of the MTC nomogram in predicting LLNM is shown in **Table 4**, **Supplementary Table S1** and **Figure 3**. The AUC of the MTC nomogram was 0.826 (95% CI, 0.762–0.891) in the training dataset, 0.816 (95% CI, 0.691–0.941) in the internal validation dataset, and 0.846 (95% CI, 0.706–0.985) in the external test dataset. Both of ROC curves and waterfall figures in **Figure 3** revealed that the MTC nomogram showed excellent prediction ability in three datasets. The MTC nomogram achieved a highest AUC in the training dataset, with an accuracy of 73.68%, a highest specificity of 76.47% and a sensitivity of 77.42%. Similarly, the AUC of the MTC nomogram was higher than other three independent risk factors in the both internal and external testing dataset, with a highest sensitivity of 87.10% in the internal testing dataset and a highest specificity of 75.00% in the external testing dataset.

**Table 4:** Predictive performance of MTC nomogram and other risk factors in the training and testing datasets.

	AUC (95% CI)	Sensitivity(%)	Specificity(%)
Training dataset			
MTC Nomogram	0.8261(95% CI: 0.7616-0.8905)	0.7619	0.7059
Gender (male)	0.6331(95% CI: 0.5556-0.7105)	0.6471	0.6190
Relationship to thyroid capsule	0.6935(95% CI: 0.6144-0.7725)	0.7353	0.6429
Ln_Ctn	0.7401(95% CI: 0.6628-0.8174)	0.9118	0.4881
Internal testing dataset			
MTC Nomogram	0.8161(95% CI: 0.6913-0.9409)	0.8710	0.6000
Gender (male)	0.571(95% CI: 0.4358-0.7061)	0.4000	0.7419
Relationship to thyroid capsule	0.6387(95% CI: 0.4942-0.7833)	0.6500	0.6774
Ln_Ctn	0.8065(95% CI: 0.6862-0.9267)	0.7500	0.7742
External testing dataset			
MTC Nomogram	0.8458(95% CI: 0.7063-0.9853)	0.8000	0.7500
Gender (male)	0.5812(95% CI: 0.4018-0.7607)	0.5630	0.6000
Relationship to thyroid capsule	0.7417(95% CI: 0.563-0.9204)	0.8125	0.7333
Ln_Ctn	0.7896(95% CI: 0.6132-0.966)	0.9375	0.6667

AUC, area under the curve; LN_Ctn, natural logarithm of serum calcitonin value.

**Figure 4:**
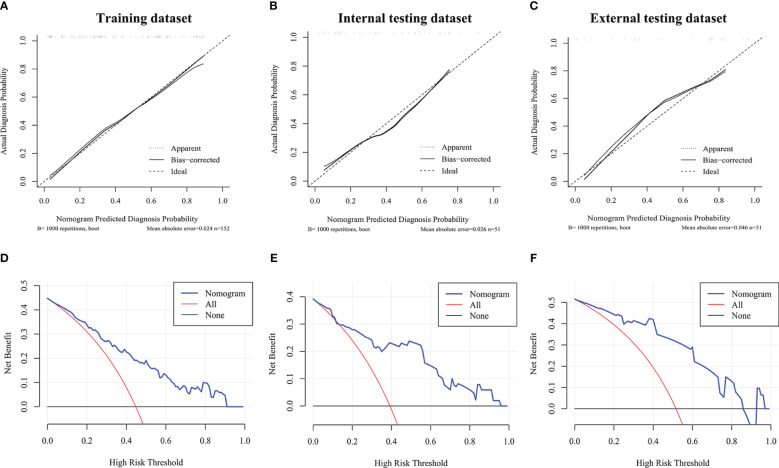
Calibration curves and decision curve analysis. **(A–C)** Calibration curve of the MTC nomogram in the training dataset and two validation datasets. **(D–F)** Decision curve analysis of the MTC nomogram in the training dataset and two validation datasets. The x-axis represents the threshold probability, and the y-axis represents the net benefit.

The calibration curves of MTC nomogram exhibited good consistency between the bias-corrected prediction and ideal reference lines with an additional 1000 bootstraps in the training and two testing datasets (**Figures 4A–C**). We also performed decision curve analysis (DCA) to compare the clinical availability and benefits of MTC nomogram and traditional US methods in estimating the risk of LLNM. The DCA curves of the MTC nomogram showed greater net benefits across a range of LLNMs risks in the three datasets than the other factors (**Figures 4D–F**).

### 3.4 Clinical application of the MTC nomogram

**Figure 5:**
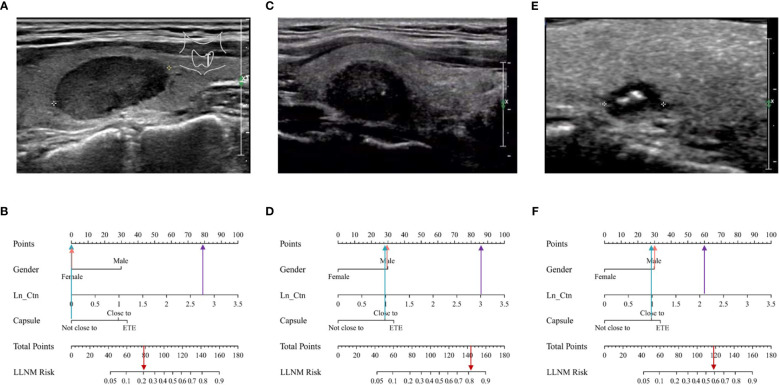
Examples of clinical application of the MTC nomogram. **(A)** Image was obtained from a 48-year-old woman with nodule in the left thyroid. **(B)** The nomogram resulted in a total score of 79 points for woman (0 points), not close to thyroid capsule (0 points), and serum Ctn was 570ng/L and Ln_Ctn=2.76 (79 points). The corresponding risk of LLNM was 0.21, and the pathological result of the nodule was MTC without LLNM. **(C)** Image was obtained from a 45-year-old man with nodule in the right thyroid. **(D)** The total points of the nomogram were 144 for man (30 points), closed to thyroid capsule (28 points), and serum Ctn was 1070ng/L and Ln_Ctn=3.03 (86 points). The corresponding risk of LLNM was 0.81, and the pathological result of the nodule was PTC with LLNM. **(E)** Image was obtained from a 44-year-old man with nodule in the left thyroid. **(F)** The total points of the nomogram were 118, for man (30 points), closed to thyroid capsule (28 points), and serum Ctn was 135ng/L and Ln_Ctn=2.13 (60 points). The corresponding risk of LLNM was 0.59, and the pathological result of the nodule was PTC with LLNM.

Representative examples of predicting the risk of LLNM in MTC patients are shown in **Figure 5**. The thyroid nodule in **Figure 5A** was obtained from a woman with a tumor on the left lobe of thyroid, far away from thyroid capsule. The maximum diameter of the tumor was 2.1cm. Her serum Ctn was 570ng/L and Ln_Ctn=2.76. The nomogram scored 79 in total, with the probability of LLNM using the nomogram model was 21% (**Figure 5B**). The nodule was MTC without LLNM according to postoperative pathological report and FNA test. The thyroid nodule in **Figure 5C** was obtained from a man with a tumor on the right lobe of thyroid, closed to thyroid capsule. His serum Ctn was 1070ng/L and Ln_Ctn=3.03. The nomogram scored 144 in total, with the risk of LLNM was 81% according to the nomogram (**Figure 5D**). The tumor was MTC with LLNM of levels II, III, IV, V and VI according to postoperative pathological report. **Figure 5E** was from a male patient with a nodule on the right thyroid, which was closed to thyroid capsule. His serum Ctn was 135ng/L and Ln_Ctn=2.13. The maximum diameter of this nodule was only 0.76cm. However, the nomogram scored 118 in total, with probability of LLNM using the nomogram model was 59% (**Figure 5F**). The nodule was MTC with LLNM of level IV according to postoperative pathological report.

## 4 Discussion

In this research, we constructed and validated the MTC nomogram based on clinical and ultrasound characteristics to predict the probability of LLNM in patients with MTC. The MTC nomogram effectively categorized patients based on their risk of LLNM, and achieved excellent performance in both internal and external testing datasets. Therefore, the preoperative probability of LLNM can be estimated individually and noninvasively. Our study has several advantages: (I) To the best of our knowledge, this is the largest scale retrospective consecutive multicenter study to construct and evaluate a nomogram to predict status of LLNM in MTC. This study included MTC patients within 10 years of Tianjin medical cancer institute and hospital, and contained the results of more than one year of follow-up for all the patients (II) Different from published studies based on radiomics, contrast enhanced ultrasound (CEUS), or TNM staging of the postoperative pathology, this novel nomogram only incorporated clinical and gray-scale US factors preoperatively, which increasing the general applicability of the model. It was particularly important for MTC patients in underdeveloped countries and regions. (III) Comparing with other machine learning (ML) models which were not visual in previous studies, the MTC nomogram we proposed had a better interpretability and maneuverability in clinical practice.

Routine measurement of basal serum Ctn is reported as an important part of the diagnosing and staging of MTC. There is a discrepancy between the different guidelines. The American Association of Clinical Endocrinologists/Associazione Medici Endocrinologi/European Thyroid Association (AACE/AME/ETA) guidelines have suggested examining CT only in subjects with a familial history of MTC and patients with cytology suggestive of MTC or undergoing surgery for goiter (13). On the other hand, according to ATA recommendations, serum Ctn measurement should not be performed routinely, mainly for cost-effectiveness reasons. Usually, preoperative diagnosis is based on cytological examination. However, Jassal et al. showed that preoperative serum Ctn measurement can improve the evaluation of MTC with indeterminate cytology and be helpful in planning surgery (14). It has been shown that, in the diagnosis of MTCs, the measurement of serum Ctn concentration has a higher sensitivity than cytological evaluation of FNA biopsy samples, so it is possible that in the future, serum Ctn concentration measurement will be used in routine diagnosis not only in Europe but also elsewhere.

Calcitonin concentration is directly proportional to the degree of progression. With a serum Ctn values of 60–100 pg/mL, there is a strong probability of the presence of a C-cell proliferative process. Zhu et al. (15) thought MTC patients with a pre-operative serum Ctn level >302.50 pg/mL were more likely to have CLNM. Some researchers indicated that the thresholds of preoperative serum calcitonin according to disease extent were 20 pg/mL for ipsilateral lateral LNM, 200 pg/mL for contralateral lateral LNM, and 500 pg/mL for distant metastases (16). Many guidelines indicated that the preoperative serum calcitonin level and ultrasound findings can be helpful for determining the need for lateral node dissection (5, 17). However, there is currently no globally uniform and standard cutoff value of serum Ctn to indicate LLNM. In our study, there is a positive correlation between preoperative serum calcitonin levels and the risk of lateral cervical lymph node metastasis, and the cutoff value of serum Ctn is 317 pg/mL. The main limitation of serum Ctn is the risk of false-positive results. For example, false-positive results of serum Ctn measurement may be caused by ectopic production of Ctn by neuroendocrine tumors. Increased serum calcitonin levels are also observed in hypercalcemia, during the use of proton pump inhibitors, in renal failure, chronic obstructive pulmonary disease and hypothyroidism (18, 19). In our study, hypothyroidism caused by Hashimoto’s thyroiditis may be a major cause of false-positive results.

Consistent with previous studies, male sex was identified as independent risk factor predicting LLNM in MTC patients in our study. But the independent predictors of LLNM in MTC are not in agreement in different researches. Jin et al. reported that only multifocal lesions and suspected LLNM in preoperative US were independent risk factors of LLNM in MTC patients (20). Zhou et al. believed that sex, tumor size, multifocality, extrathyroidal extension, and distant metastasis were identified to be associated with LLNM in MTC (21). Wu et al. showed that patients with MTC (≤1cm) have a lower risk of lateral lymph node metastasis than those with tumor size greater than 1cm (22). However, multifocality and tumor size were not risk factors of LLNM in our study, which may need to be further verified by prospective researches of patients.

Previous studies have found that extrathyroidal extension often reflects the tumor aggressiveness of MTC. Once the malignant tissue infiltrates the capsule, it is easy to enter the lymphatic circulation system, which makes it extremely prone to lymph nodes. Studies showed that extrathyroidal extension was also an independent risk factor for LLNM and recurrence (21, 23). However, our result showed that not only extrathyroidal extension but the position of thyroid nodule close to capsule (≤2mm) was also an independent predictive indicator to LLNM. When the nodule was near the capsule, the thyroid capsule might have received invasion.

Previous studies have demonstrated that nomograms have been useful in predicting LLNM risk in PTC patients (24, 25), but there were relatively few reports for predicting LLNM risk in MTC. Previous nomograms for estimating LLNM of MTC were constructed basing on different risk parameters, such as suspected LLNM in preoperative ultrasound and distant metastasis (20). However, the existence of occult LLNM is not easily detected in ultrasound and the incidence of distant metastases is relatively low, making this index not applicable for the vast majority of MTC. Some scholars predicted LLNM in MTC by the SEER database, but it lacked an important data of calcitonin, which benefited a lot in predicting LLNM in MTC (21). Besides, previous studies of MTC are all single-center studies with a small number of samples (3), so the applicability is limited to a certain extent.

There are several limitations in the present study. First, it was designed retrospectively, and the assessment of static US images has an inherent limitation to the precision of US interpretation. Second, this study did not distinguish between the subtypes of MTC. Whether the model is applicable to hereditary MTC requires further verification. Third, given that LND was only performed in patients with high suspicion of LLNM based on preoperative imaging and FNAC, microscopic metastases might be overlooked. Last, the external validation dataset contains a small number of samples, and further confirmation with a large sample and prospective clinical trial is needed in the future.

## 5 Conclusions

In conclusion, we comprehensively identified individual predictive factors of LLNM for patients with MTC, including gender (male), the relationship with the thyroid capsule and serum Ctn. The nomograms were proved to be accurate, efficient, and clinically beneficial in preoperative guiding the clinical diagnosis and treatment process of MTC patients.

## Data availability statement

The raw data supporting the conclusions of this article will be made available by the authors, without undue reservation.

## Ethics statement

The studies involving humans were approved by The Ethics Committee of Tianjin Medical University Cancer Institute and Hospital. The studies were conducted in accordance with the local legislation and institutional requirements. The ethics committee/institutional review board waived the requirement of written informed consent for participation from the participants or the participants’ legal guardians/next of kin because Patient consent was waived due to the retrospective nature of the study. Written informed consent was obtained from the individual(s) for the publication of any potentially identifiable images or data included in this article.

## Author contributions

JLZ: Writing – review & editing, Writing – original draft, Funding acquisition, Conceptualization. TG: Writing – original draft, Methodology. SG: Writing – original draft, Methodology, Funding acquisition. LC: Writing – original draft, Validation, Methodology, Data curation. JZ: Writing – original draft, Data curation. XQW: Writing – original draft, Data curation. XW: Writing – review & editing, Writing – original draft, Project administration, Conceptualization.
