# Supplementary material for: A practical nomogram for preoperatively predicting lateral cervical lymph node metastasis in medullary thyroid carcinoma: a dual-center retrospective study
Source: Front Endocrinol (Lausanne). 2024 Jul 26;15:1349853. doi: 10.3389/fendo.2024.1349853 (PMC11310050; doi:10.3389/fendo.2024.1349853)
Supplement: Supplementary file 1 [file DataSheet_1.docx]

Supplementary Material

# Supplementary Data

Supplementary Material 1:

**FNAC and surgical strategy**

US-guided FNAC was performed by radiologists with more than 15 years of experience in thyroid FNAC with at least three repeated aspirations were performed in different directions for each nodule, using 22-gauge needles. According to US-guided FNAC, all patients enrolled in this study were confirmed as Bethesda Categories V or VI.

Cervical lymph nodes with spherical shape, normal echo of lymphatic hilum disappeared, cystic components, microcalcifications or peripheral vascularity were suspected to be metastatic. FNAC test were also conducted on the most suspicious lateral cervical lymph nodes before surgery to confirm the pathological diagnosis.

Total thyroidectomy or thyroid lobectomy were performed on all patients, along with central neck dissection (CND) according to the Chinese guidelines for diagnosis and treatment of differentiated thyroid carcinoma. According to the ATA and Chinese guidelines, only patients with highly suspected metastatic lateral neck lymph nodes based on preoperative imaging data and FNAC, underwent LND, which comprised of removal of the lateral lymph nodes from level II to V, while preserving the internal jugular vein, spinal accessory nerve, or sternocleidomastoid muscle.

Supplementary Material 2:

**Ultrasound characteristics evaluating**

The following characteristics were evaluated for all selected thyroid nodules: maximum diameter of tumor (tumor size), position, mulifocality, composition, echogenicity, margin, shape (A/T≥1 or <1), and microcalcification (punctate echogenic foci without shadowing). Vascularization (blood flow) was classified according to the Adler grade of blood flow from 0 to 3. In addition, the diagnosis of Hashimoto's thyroiditis is based on ultrasound findings and serological tests, including positive thyroid peroxidase antibodies (TPOAb) and positive thyroid globulin antibodies (TG-Ab). Hashimoto's thyroiditis manifests as uneven echogenicity of the thyroid parenchyma on ultrasonography, with a few or multiple lamellar hypoechoic areas showing grid-like changes. The adjacent relationship with thyroid capsule were evaluated on the basis of US images. The location of nodule more than 2mm away from thyroid capsule was defined as not close to capsule. The nodule location less than 2mm from the thyroid capsule was defined as close to the capsule. The nodule invading the capsule or breakthrough the thyroid capsule was defined as extrathyroidal extension (ETE).

# Supplementary Table 1

| Table S1. Predictive performance of MTC nomogram in the training and testing datasets. | | | | | | |  |
| --- | --- | --- | --- | --- | --- | --- | --- |
|  | AUC (95% CI) | Accuracy (95% CI) | Sensitivity | Specificity | PPV | NPV | |
| **Training dataset** | **0.826**  **(95% CI: 0.7616-0.8905)** | 0.7368  (95% CI : 0.6593,-0.8049) | 76.19% | 70.59% | 76.19% | 70.59% | |
| **Internal testing dataset** | **0.816**  **(95% CI: 0.6913-0.9409)** | 0.7647  (95% CI : 0.6251-0.8721) | 87.10% | 60.00% | 77.14% | 75.00% | |
| **External testing dataset** | **0.846**  **(95% CI: 0.7063-0.9853)** | 0.7742  (95% CI:0.589-0.9041) | 80.00% | 75.00% | 75.00% | 80.00% | |

Note—PPV, Positive predictive value; NPV, Negative predictive value
